# Genome-wide detections for runs of homozygosity and selective signatures reveal novel candidate genes under domestication in chickens

**DOI:** 10.1186/s12864-024-10349-4

**Published:** 2024-05-16

**Authors:** Xiaodong Tan, Lu Liu, Jie Dong, Minjie Huang, Jiawen Zhang, Qinghai Li, Huanhuan Wang, Lijuan Bai, Ming Cui, Zhenzhen Zhou, De Wu, Yun Xiang, Weifen Li, Deqian Wang

**Affiliations:** 1 Institute of Animal Husbandry and Veterinary Science, Zhejiang Academy of Agricultural Sciences, Hangzhou, 310021 China; 2 College of Animal Sciences, Zhejiang University, Hangzhou, Zhejiang 310058 China; 3 Jinhua Jinfan Feed Co., Ltd, Jinhua, Zhejiang 321000 China; 4 Animal Husbandry Institute, Hangzhou Academy of Agricultural Sciences, Hangzhou, 310024 China; 5 Zhejiang Animal Husbandry Technology Extension and Breeding Livestock and Poultry Monitoring Station, Hangzhou, 310020 China; 6 Postdoctoral Research Station, Jinhua Development Zone, Jinhua, Zhejiang 321000 China

**Keywords:** Indigenous chickens, Genomic inbreeding, Runs of homozygosity, Selective signature, Transcriptome, *VSTM2A*

## Abstract

**Background:**

Indigenous chickens were developed through a combination of natural and artificial selection; essentially, changes in genomes led to the formation of these modern breeds via admixture events. However, their confusing genetic backgrounds include a genomic footprint regulating complex traits, which is not conducive to modern animal breeding.

**Results:**

To better evaluate the candidate regions under domestication in indigenous chickens, we considered both runs of homozygosity (ROHs) and selective signatures in 13 indigenous chickens. The genomes of Silkie feather chickens presented the highest heterozygosity, whereas the highest inbreeding status and ROH number were found in Luhua chickens. Short ROH (< 1 Mb), were the principal type in all chickens. A total of 291 ROH islands were detected, and QTLdb mapping results indicated that body weight and carcass traits were the most important traits. An ROH on chromosome 2 covering *VSTM2A* gene was detected in 12 populations. Combined analysis with the Tajima’s D index revealed that 18 genes (e.g., *VSTM2A*, *BBOX1*, and *RYR2*) were under selection and covered by ROH islands. Transcriptional analysis results showed that *RYR2* and *BBOX1* were specifically expressed in the heart and muscle tissue, respectively.

**Conclusion:**

Based on genome-wide scanning for ROH and selective signatures, we evaluated the genomic characteristics and detected significant candidate genes covered by ROH islands and selective signatures. The findings in this study facilitated the understanding of genetic diversity and provided valuable insights for chicken breeding and conservation strategies.

**Supplementary Information:**

The online version contains supplementary material available at 10.1186/s12864-024-10349-4.

## Background

After domestication from the ancestral red jungle fowl (RJF), hundreds of indigenous chicken breeds have been cultivated and distributed around the world [1, 2]. These include meat-type chickens (e.g., Xiaoshan chicken, Pudong chicken), egg-type chickens (e.g., Xianju chicken, Baier Yellow chicken), gamecocks (e.g., Turpan Game chicken), and ornamental chickens (e.g., Silkie feather chicken). These chickens harbor considerable phenotypic diversity and genetic variation, provide diversified animal products and play an important role in cultivating new breeds/lines in modern chicken breeding systems [3]. Indigenous chickens are often subjected to both domestication and selection; however, the selection effect is rather weak, and more genetic diversity is found in indigenous chickens than in commercial broilers [4–6]. Genetic and genomic studies on these chickens are relatively limited. Therefore, most indigenous chickens are classified as showing undeveloped or conservation status. The lack of effective methods and bases for the accurate evaluation of animal conservation and development and utilization of genetic resources is nonnegligible.

Whole-genome sequencing studies provide the possibility of evaluating the genetic structure and population diversity using the single nucleotide polymorphisms (SNPs) across the genome. Furthermore, calculated runs of homozygosity (ROHs; long continuous homozygous stretches composed of two identical haplotypes in each individual) are more useful for elucidating inbreeding and detecting selection signatures in different populations [7, 8]. This approach has been reported in cattle [9], pigs [10], goats [11], and chickens [12]. The inbreeding coefficient based on ROH (F_ROH_) is more accurate and more closely represents real inbreeding at the population and individual levels, and a long ROH is more likely to be a result of recent inbreeding than a shorter ROH [13]. Wang et al. compared the ROH distribution in one selected and one natural chicken line, and ROH length and number were significantly greater in the selected line than in the natural line [12]. Yuan et al. calculated ROH numbers and lengths in five Tibetan chickens and identified a common candidate region harboring the *AMY2A, NTNG1,* and *VAV3* genes [14]. Nothnagel et al. defined an ROH island as a genomic interval with a high incidence of homozygosity across individuals, which provides novel insights into the selective signatures within populations due to linkage disequilibrium [15]. Zhang et al. detected 191 ROH islands in commercial, local, game, and wild chickens, and these regions were shown to be involved in egg production, growth, and Silkie feather traits [16]. The detection of ROH islands improves the understanding of molecular mechanisms related to environmental adaptation. Yuan et al. revealed that *BDNF*, *CCDC34*, *LGR4*, etc., which are common genes in ROH islands, play essential roles in high-altitude adaptation [14]. Fedorova et al. further suggested that *ADIPOQ*, *GCGT*, *TRPM2*, etc., which are genes found in specific ROH islands, were correlated with cold adaptation in chickens in the early postnatal period [17].

In chickens, ROH analysis has been used to assess genome diversity and the inbreeding status of chicken breeds [18, 19] and aids in the design and review of effective breed conservation projects. ROH indicate recent selection or bottlenecks and are more likely to be focused on regions with low recombination rates [20]. Here, we evaluated genome diversity and ROH islands in multiple indigenous chicken breeds and annotated ROH islands using enrichment and quantitative trait locus (QTL) databases, and we present the expression profiles of candidate genes in common ROH islands combined with transcriptomic characteristics. These results are expected to provide valuable information for the conservation and development of chicken breeds and to facilitate the understanding of the molecular mechanism of domestication.

## Methods

### Experimental animals

A total of 209 chickens from 13 chicken breeds were used in this study. These 13 breeds included Baier chicken (BE, *n* = 10), Beijing You chicken (BJY, *n* = 20), Dongxiang Black chicken (DXB, *n* = 20), Jiangshan white-feathered chicken (JSW, *n* = 19), Luhua chicken (LH, *n* = 20), Longyou chicken (LY, *n* = 10), Silkie feather chicken (SF, *n* = 10), Songyang Jin chicken (SYJ, *n* = 17), Wenling chicken (WL, *n* = 20), Xianju chicken (XJ, *n* = 14), Xiaoshan chicken (XS, *n* = 20), Xiaoxiang chicken (XX, *n* = 19), and Yandang chicken (YD, *n* = 10), which were provided by Animal Husbandry Institute Hangzhou Academy of Agricultural Sciences and Zhejiang Animal Husbandry Technology Extension and Breeding Livestock and Poultry Monitoring Station.

### Sample collection and DNA extraction

A total of 1 ml blood from the wing vein of each individual was collected and stored at -20°C for subsequent DNA extraction. Genomic DNA was extracted from blood samples (*n* = 209) using the phenol‒chloroform method. Solutions of extracted DNA were quality controlled by measuring DNA degradation and contamination using agarose gel electrophoresis, and purity and DNA concentration using NanoPhotometer-N50 (Implen, Schatzbogen, Germany).

### Library preparation and whole-genome sequencing

A library was generated using the NEB Next® Ultra™ DNA Library Prep Kit (NEB, USA), including fragmentation, end-polishing, A-tailing, adaptor ligation, PCR amplification, etc. The 5' ends of the qualified libraries were phosphorylated and cyclized; then, the libraries were subjected to rolling loop amplification; and the DNA nanospheres (DNBs) were finally loaded into a flow cell and sequenced on a DNBSEQ-T7 platform to generate paired-end reads of 150 bp in length. In all, 3.67 Tb raw data were generated to an average depth of 21 × .

### Reads mapping, variants calling, and quality control

The raw reads were first trimmed using FASTP v0.21 [21], and clean reads were produced for genome mapping. The standards for quality control were reported previously [5]. Next, we aligned the filtered reads of all individuals to the reference genome (GRCg7b, https://ftp.ncbi.nlm.nih.gov/genomes/all/GCF/016/699/485/GCF_016699485.2_bGalGal1.mat.broiler.GRCg7b/GCF_016699485.2_bGalGal1.mat.broiler.GRCg7b_genomic.fna.gz) using the MEM algorithm in Burrows‒Wheeler Aligner (BWA) v0.7.17 [22]. The file conversion, sorting, and indexing of the genome mapping results were completed with SAMtools v1.12 [23]. PCR duplicates were marked and removed using PICARD v2.26. Then, SNP calling analysis was conducted in line with the recommended joint calling pipeline from Genome Analysis Toolkit Kit (GATK) v4.2.2 [24], including HaplotypeCaller, CombineGVCFs, and GenotypeGVCFs functions. In addition, we removed SNPs using GATK with the following standards: quality score < 30.0, QualByDepth < 2.0, FisherStrand > 60.0, RMSMappingQuality < 40.0, StrandOddsRatio > 3.0, MappingQualityRankSumTest < -12.5, and ReadPosRankSum < -8. The reserved SNPs were further filtered according to allele frequency and sequencing depth using VCFtools (–max-alleles 2 –min-alleles 2 –min-meanDP 3 –maf 0.01). A total of 19,820,641 biallelic SNPs were retained for the following analysis. Missing alleles were imputed by Beagle 5.1 software [25].

### Genome heterozygosity and inbreeding analysis

The filtered SNPs were further used to calculate observed heterozygosity (Ho) and expected heterozygosity (He) using PLINK v1.9 software [26] with the ‘-hardy’ option. Genomic inbreeding coefficients based on genomic SNP-by-SNP (F_GRM_), excess homozygosity (F_HOM_), and uniting gametes (F_UNI_) are universally accepted in farm animals [27] and were calculated in this study as ${F}_{HOM}=\frac{\left(O-E\right)}{\left(L-E\right)}$, where $O$ is the number of observed homozygotes, $E$ is the number of expected homozygotes, and $L$ is the number of genotyped autosomal SNPs. Genomic SNP-by-SNP inbreeding (FGRM) and the correlation between uniting gametes (F_UNI_) were estimated in GCTA v1.93.2 software. The formula was as follows: ${F}_{GRM}=\frac{1}{m}\sum_{i=1}^{N}\left(\frac{{\left[{x}_{i}-E\left({x}_{i}\right)\right]}^{2}}{2{p}_{i}\left(1-{o}_{i}\right)}-1\right)$, ${F}_{UNI}=\frac{{x}^{2}-1+2{p}_{i}{x}_{i}+2{p}_{i}^{2}}{2{p}_{i}\left(1-{p}_{i}\right)}$, where m is SNP number and ${x}_{i}$ and ${p}_{i}$ are the copy number and frequency of the reference allele of the i^th^ SNP, respectively.

### ROH detection and statistics

ROH fragments were scanned in each individual using PLINK v1.9 according to the following options: 1) the minimum length of an ROH was 300 kb (–homozyg-kb 300); 2) more than 50 SNPs were detected in an ROH (–homozyg-snp 50) and sliding windows (–homozyg-window-snp 50); 3) fewer than 5 missing SNPs (–homozyg-window-missing 5) and 3 heterozygous SNPs (–homozyg-window-het 3) were found in each sliding window; 4) the minimum density was 1 SNP per 50 kb (–homozyg-density 50); and 5) the maximum gap between two consecutive SNPs was less than 1000 kb (–homozyg-gap 1000) [5]. ROH numbers were counted, and ROH were classified into four groups (< 1 Mb, 1 ~ 2 Mb, 2 ~ 3 Mb, > 3 Mb) based on length. In addition, the total length of ROH in each individual and autosome covered by SNPs in this dataset was calculated, and the genomic inbreeding coefficient based on ROH (F_ROH_) was estimated as follows: ${F}_{ROH}=\frac{{\sum }_{i}{L}_{{ROH}_{i}}}{{L}_{auto}}$, where ${L}_{{ROH}_{i}}$ is the ROH length of the i^th^ individual, and ${L}_{auto}$ is the genome length of autosome [28].

### ROH island detection, annotation, enrichment analysis

To detect the candidate regions related to selection and domestication, we estimated the ROH incidence according to the percentage of animals with a SNP within an ROH segment for a given population [15, 29]. To explore more putative regions, we extended the threshold applied in Purfield et al.’s report [30], and the top 0.5% of SNPs occurring in ROH were considered to form ROH islands. The ROH incidence among these 13 breeds was higher than 30%. Thereafter, we annotated these common ROH islands based on the Gallus gallus 7.0 assembly using Ensembl BioMart tools to identify the candidate genes [31]. Kyoto Encyclopedia of Genes and Genomes (KEGG) and Gene Ontology (GO) enriched analysis of annotated genes were conducted in the Omicshare (https://www.omicshare.com/) and KOBAS platform (http://kobas.cbi.pku.edu.cn/) [32], respectively. And a *p* value of 0.05 was set as the threshold of significance for GO terms and KEGG pathways. Additionally, chicken QTLdb (https://www.animalgenome.org/cgi-bin/QTLdb/GG/browse) [33] was employed to detect the overlap between current QTLs and common ROH islands and to determine putative physiological functions under selection in each breed.

### Identification of common ROH island and frequency comparison

The common ROH islands in most chickens could plausibly be associated with human selection or domestication. To identify selection- or domestication-related candidate genes, we first counted the overlapping ROH islands in each breed and annotated them as mentioned above [29], and only protein-coding genes were summarized for each breed. Then, only genes identified in more than 7 chicken breeds (> 50%) were considered candidate genes. The frequency of ROH in regions around these genes in each breed has been calculated [14]. In addition, the whole-genome variants of chicken ancestors (RJF) were obtained from Wang et al. [34], and similar results were incorporated as unselected controls to determine whether the candidate genes were selected in indigenous chickens.

### Selective sweep analysis and gene annotation

To explore selection signatures in the ROH islands of indigenous chickens, the Tajima’s *D* index with a window of 2 kb was calculated using VCFtools v0.1.13 software [35]. A similar calculation was performed for the RJF population. Only the genomic regions defined as ROH islands were used to perform selection signature analysis. The genomic regions were regarded as putative selected regions when a negative Tajima’s *D* value in indigenous chickens that was lower than that of RJFs was identified. The genes with high ROH island frequencies that overlapped with putative selected genes were defined as candidate genes in this study.

### Expression profile of candidate genes in multiple tissues

Published raw RNA-seq data of 64 samples of six tissues (breast muscle, thigh muscle, heart, lung, liver, and abdominal fat) collected from BJY chickens at day 1 and day 42 were used [5]. More than 10 Gb of raw reads per sample were generated, and the bioinformatic analysis pipeline (trimming (Trimmomatic v0.39) [36], genome mapping (HISAT2 v2.2.1) [37], assembly-merge-assembly-counting (StringTie v2.1.6) [38], counting and normalization (DESeq2) [39]) was implemented as previously reported. The raw gene count number was obtained using the Python script provided by StringTie (l = 150). The final gene expression value was normalized using the DESeq2 package [39]. The expression profiles of candidate genes were visualized in the R environment.

## Results

### Statistics of genomic heterozygosity and inbreeding

**Fig. 1:**
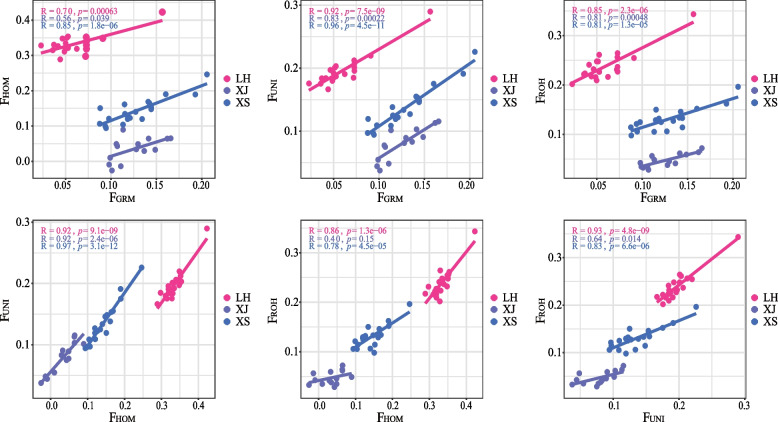
Correlation of genomic inbreeding in indigenous chickens. The scatter plot pre-sented the results of LH, XJ, and XS populations, similar results of other populations was shown in Fig. S1. LH, Luhua chicken; XJ, Xianju chicken; XS, Xiaoshan chicken

Observed heterozygosity (Ho) and expected heterozygosity (He) were estimated for the 13 chicken populations using all SNPs (Table 1). Ho ranged from 0.26 to 0.32, and the estimates of He ranged from 0.27 to 0.31. The Silkie feather chickens (SF) presented the highest genome heterozygosity (Ho = 0.32, He = 0.31). To determine the inbreeding coefficient, we estimated the inbreeding coefficients based on genomic SNP-by-SNP (F_GRM_), excess homozygosity (F_HOM_), and uniting gametes (F_UNI_), and FROH to assess the inbreeding status of each population (Table 1). In the different populations, the inbreeding coefficient ranged from 0.02 to 0.33 (F_GRM_: 0.05 ~ 0.20, F_HOM_: 0.02 ~ 0.33, F_UNI_: 0.04 ~ 0.20, F_ROH_: 0.02 ~ 0.24). The highest inbreeding status was observed in the Luhua chickens (LH) based on the F_HOM_, F_UNI_, and F_ROH_ estimates, while the lowest inbreeding status was observed in the Baier chickens (BE) and Longyou chickens (LY). A significant positive correlation among these inbreeding coefficients was found in most populations (Fig. 1, Fig. S1). However, some correlations, such as the correlation between F_ROH_ and F_HOM_ in Xianju chickens (XJ) (Fig. 1), did not reach the significance threshold, and the partial inbreeding coefficient associations in BE, LY, Wenling chickens (WL), and Beijing You chickens (BJY) were also rather weak and insignificant (Fig. S1).

**Table 1:** Genomic heterozygosity and inbreeding for each chicken population

Population^a^	Ho	He	F_GRM_	F_HOM_	F_UNI_	F_ROH_
BE	0.31 ± 0.18	0.29 ± 0.15	0.07 ± 0.01	0.02 ± 0.01	0.04 ± 0.01	0.02 ± 0.004
BJY	0.28 ± 0.18	0.28 ± 0.16	0.09 ± 0.02	0.13 ± 0.03	0.11 ± 0.02	0.10 ± 0.02
DXB	0.30 ± 0.18	0.29 ± 0.15	0.05 ± 0.03	0.16 ± 0.04	0.11 ± 0.03	0.11 ± 0.03
JSW	0.30 ± 0.18	0.30 ± 0.15	0.06 ± 0.03	0.16 ± 0.04	0.11 ± 0.04	0.11 ± 0.03
LH	0.31 ± 0.18	0.30 ± 0.15	0.06 ± 0.03	0.33 ± 0.03	0.20 ± 0.03	0.24 ± 0.03
LY	0.31 ± 0.18	0.29 ± 0.15	0.05 ± 0.01	0.03 ± 0.02	0.04 ± 0.01	0.03 ± 0.01
SF	0.32 ± 0.18	0.31 ± 0.14	0.15 ± 0.02	0.10 ± 0.03	0.13 ± 0.02	0.09 ± 0.02
SYJ	0.28 ± 0.18	0.28 ± 0.15	0.15 ± 0.03	0.02 ± 0.03	0.08 ± 0.03	0.03 ± 0.01
WL	0.26 ± 0.17	0.27 ± 0.16	0.11 ± 0.03	0.04 ± 0.03	0.07 ± 0.02	0.05 ± 0.03
XJ	0.28 ± 0.18	0.28 ± 0.15	0.12 ± 0.02	0.03 ± 0.03	0.08 ± 0.03	0.05 ± 0.01
XS	0.30 ± 0.17	0.30 ± 0.15	0.13 ± 0.03	0.14 ± 0.04	0.14 ± 0.03	0.13 ± 0.02
XX	0.29 ± 0.18	0.28 ± 0.15	0.20 ± 0.06	0.02 ± 0.06	0.11 ± 0.06	0.08 ± 0.04
YD	0.31 ± 0.18	0.31 ± 0.14	0.15 ± 0.03	0.06 ± 0.02	0.11 ± 0.02	0.09 ± 0.02

^a^*BE* Baier chicken, *BJY* Beijing You chicken, *DXB* Dongxiang Black chicken, *JSW* Jiangshan white-feathered chicken, *LH* Luhua chicken, *LY* Longyou chicken, *SF* Silkie feather chicken, *SYJ* Songyang Jin chicken, *WL* Wenling chicken, *XJ* Xianju chicken, *XS* Xiaoshan chicken, *XX* Xiaoxiang chicken, *YD* Yandang chicken

### ROH identification and distribution across the whole genome

**Fig. 2:**
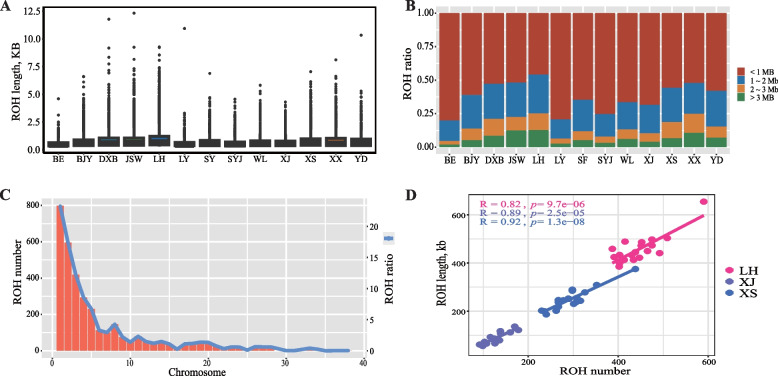
Statistics for ROH number, length, and distribution in genome. **A** The length distribution of each ROH in indigenous chickens. **B** Summary for ROH classification based on segment length (< 1 Mb, 1 ~ 2 Mb, 2 ~ 3 Mb, > 3 Mb). **C** The average number of ROH per chromosome (bars) and the average percentage of each chromosome covered by ROH (lines) in indigenous chickens. **D** The correlation between ROH length and number in LH, XJ, and XS populations. LH, Luhua chicken; XJ, Xianju chicken; XS, Xiaoshan chicken

The ROH identified in each chicken breed are shown in Table 2. A total of 790 ~ 8,883 ROH were identified in the 13 populations (Table S1), and the average number of ROH ranged from 79.0 to 444.2. The ROH number was less than 100 in BE (79.0) and LY (83.7), and the genome of LH chickens harbored the most ROH (average 444.2). The total length of ROH in each bird ranged from 22.7 Mb to 226.0 Mb, while the average length of ROH in an individual ranged from 591.2 Kb to 1019.1 Kb (Table S1). Similar to the results for ROH numbers, relatively small ROH were observed in BE, and large ROH were found in LH chickens. We also observed that the genome of Jiangshan white-feathered chickens (JSW) harbored the longest ROH, with a length of over 12.0 Mb (Fig. 2A). The ROH were classified into 4 groups based on their length, and short ROH (< 1 Mb) were the main ROH type in the 13 populations, accounting for 67.9% to 89.9% of all ROH (Fig. 2B). As shown in Fig. 2C, the majority of ROH detected in the 13 populations were in the macrochromosomes (GGA1 to GGA5) with a ratio > 5%. Additionally, a high correlation between ROH number and length was detected in Xiaoshan chickens (XS), XJ, LH (*r* = 0.82 ~ 0.92) and other populations (*r* = 0.78 ~ 0.98) (Fig. 2D, Fig. S2). The highest correlation was found in Xiaoxiang chickens (XX) (*r* = 0.98) (Fig. S2).

**Table 2:** Summary of ROH number and length distribution in genome

Population	Number of ROH	Average number of ROH	Total length of ROH (Mb)	Average length of ROH (Kb)	ROH length distribution^1^			
(n)^a^					< 1 Mb	1 Mb—2 Mb	2 Mb—3 Mb	≥ 3 Mb
BE (10)	790	79.0 ± 13.8	22.7 ± 4.4	591.2 ± 69.3	476.9 ± 168.1	1,363.0 ± 284.1	2,331.1 ± 106.4	3,871.2 ± 851.7
					-89.90%	-8.60%	-1.00%	-0.50%
BJY (20)	4,971	248.6 ± 34.9	96.0 ± 17.4	777.8 ± 57.9	519.4 ± 181.5	1,361.8 ± 281.8	2,425.4 ± 290.9	3,817.1 ± 892.4
					-78.50%	-16.20%	-3.70%	-1.60%
DXB (20)	4,750	237.5 ± 40.7	106.3 ± 31.1	890.9 ± 117.4	527.3 ± 189.1	1,374.2 ± 259.1	2,434.0 ± 268.6	4,322.4 ± 1,552.6
					-73.10%	-18.10%	-5.90%	-2.90%
JSW (20)	4,470	235.3 ± 49.3	108.0 ± 29.9	918.5 ± 95.5	526.8 ± 186.1	1,356.2 ± 270.8	2,387.8 ± 269.6	4,278.3 ± 1,450.7
					-73.00%	-18.00%	-4.70%	-4.30%
LH (20)	8,883	444.2 ± 48.5	226.0 ± 28.7	1,019.1 ± 75.1	553.6 ± 193.8	1,399.6 ± 282.6	2,463.9 ± 288.3	4,290.2 ± 1,204.3
					-67.90%	-21.40%	-6.10%	-4.70%
LY (10)	837	83.7 ± 12.8	25.9 ± 8.8	618.9 ± 125.1	483.8 ± 170.5	1,437.7 ± 304.3	2,577.5 ± 221.2	5,795.7 ± 3,990.2
					-89.70%	-8.10%	-1.40%	-0.70%
SF (10)	2,449	244.9 ± 42.9	88.4 ± 21.2	726.7 ± 49.1	506.8 ± 175.7	1,340.1 ± 247.7	2,418.4 ± 298.8	3,669.8 ± 745.3
					-80.90%	-14.70%	-2.80%	-1.60%
SYJ (17)	1,716	100.9 ± 16.3	31.4 ± 7.4	630.5 ± 101.2	486.5 ± 173.7	1,329.5 ± 266.8	2,480.5 ± 326.1	3,604.3 ± 587.1
					-87.50%	-9.80%	-1.70%	-0.90%
WL (20)	2,669	133.5 ± 49.1	47.9 ± 24.7	693.4 ± 115.7	498.5 ± 177.8	1,348.3 ± 289.9	2,331.8 ± 251.8	3,921.7 ± 679.6
					-82.50%	-12.70%	-2.90%	-1.90%
XJ (14)	1,813	129.5 ± 26.2	44.6 ± 12.8	683.2 ± 94.2	496.3 ± 174.5	1,352.5 ± 262.0	2,447.1 ± 297.2	3,566.8 ± 398.1
					-83.30%	-12.90%	-2.60%	-1.20%
XS (20)	5,836	291.8 ± 46.2	123.3 ± 21.8	848.5 ± 60.2	531.6 ± 187.2	1,355.0 ± 269.7	2,372.8 ± 277.0	4,037.3 ± 890.7
					-75.20%	-17.20%	-5.40%	-2.20%
XX (19)	3,114	163.9 ± 71.3	74.7 ± 41.1	883.0 ± 152.5	517.0 ± 187.9	1,410.0 ± 285.0	2,379.9 ± 262.1	4,094.8 ± 1,051.4
					-73.40%	-16.20%	-6.60%	-3.70%
YD (10)	2,236	223.6 ± 35.7	89.6 ± 17.3	818.1 ± 60.9	523.8 ± 187.0	1,366.6 ± 281.8	2,388.8 ± 308.3	3,856.7 ± 1,450.0
					-76.40%	-17.6	-3.60%	-2.30%

^a^*BE* Baier chicken, *BJY* Beijing You chicken, *DXB* Dongxiang Black chicken, *JSW* Jiangshan white-feathered chicken, *LH* Luhua chicken, *LY* Longyou chicken, *SF* Silkie feather chicken, *SYJ* Songyang Jin chicken, *WL* Wenling chicken, *XJ* Xianju chicken, *XS* Xiaoshan chicken, *XX* Xiaoxiang chicken, *YD* Yandang chicken

^1^ROH length was presented as mean ± standard deviation, the ROH ratio was provided in the bracket

### ROH island detection, enrichment analysis, and QTL mapping

**Fig. 3:**
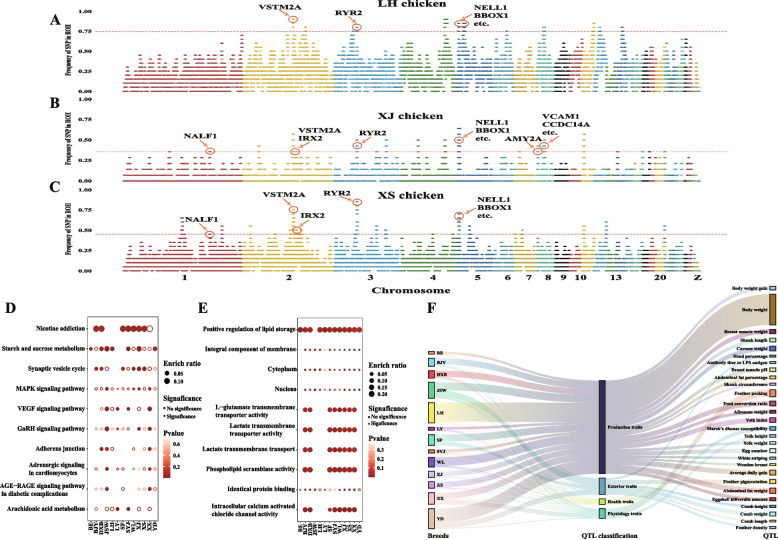
Identification, annotation, and enrichment analysis based on ROH islands. **A**-**C** Identification of ROH islands in LH, XJ, and XS populations, respectively. The red line indicated the threshold of ROH island in different populations. The annotated genes were identified by common ROH islands. **D** Top enriched pathways in the 13 indigenous chickens. **E**–**G** KEGG enrichment results based on the genes located within ROH islands in LH, XJ, and XS populations, respectively. **H** Top enriched GO terms in the 13 indigenous chickens. **I**-**K** GO enrichment results based on the genes located within ROH islands in LH, XJ, and XS populations, respectively. **L** QTL mapping based on the ROH islands in each population. BE, Baier chicken; BJY, Beijing You chicken; DXB, Dongxiang Black chicken; JSW, Jiangshan white-feathered chicken; LH, Luhua chicken; LY, Longyou chicken; SF, Silkie feather chicken; SYJ, Songyang Jin chicken; WL, Wenling chicken; XJ, Xianju chicken; XS, Xiaoshan chicken; XX, Xiaoxiang chicken; YD, Yandang chicken

ROH incidence in the 13 populations was calculated, and the top 0.5% of ROH was set as the threshold for defining ROH islands. 14, 18, and 27 merged ROH islands were detected in LH, XJ, and XS chickens, respectively (Fig. 3A-C, Fig. S3, Table S2). A total of 291 ROH islands were identified in the 13 populations (4 ~ 58 in each population, Table S2). The annotation results for these ROH islands were obtained from the Ensembl database. A total of 1,878 nonredundant protein-coding genes and long noncoding RNAs (lncRNAs) were distributed around these ROH islands in the 13 populations, and 48 ~ 484 genes were annotated in each population (Table S3). In the JSW population, the most annotated genes (484) were identified in 34 ROH islands, while the fewest genes (48) were found in the LY population. KEGG enrichment analysis showed that a total of 56 pathways were enriched based on the results from 13 populations, and we found that the starch and sucrose metabolism was significantly enriched in LH, XJ, XS and other populations (Fig. 3D, Fig. S4, Table S4). GO analysis revealed that positive regulation of lipid storage was enriched in all populations except JSW, and cytoplasm, integral component of membrane, and nucleus were also identified in over 10 populations (Fig. 3E, Fig. S5, Table S5). Additionally, the 4 ~ 58 ROH islands found in each chicken breed were mapped to the chicken QTL database (Table S6). A total of 52 ~ 382 QTLs were mapped in each population, and body weight was the leading annotated trait and common QTL (Fig. 3F, Fig. S6). Growth, carcass traits, and feed efficiency-related traits were the most enriched QTLs and may have been selected to serve human preferences.

### Identification for candidate genes based on ROH islands with high frequency

**Fig. 4:**
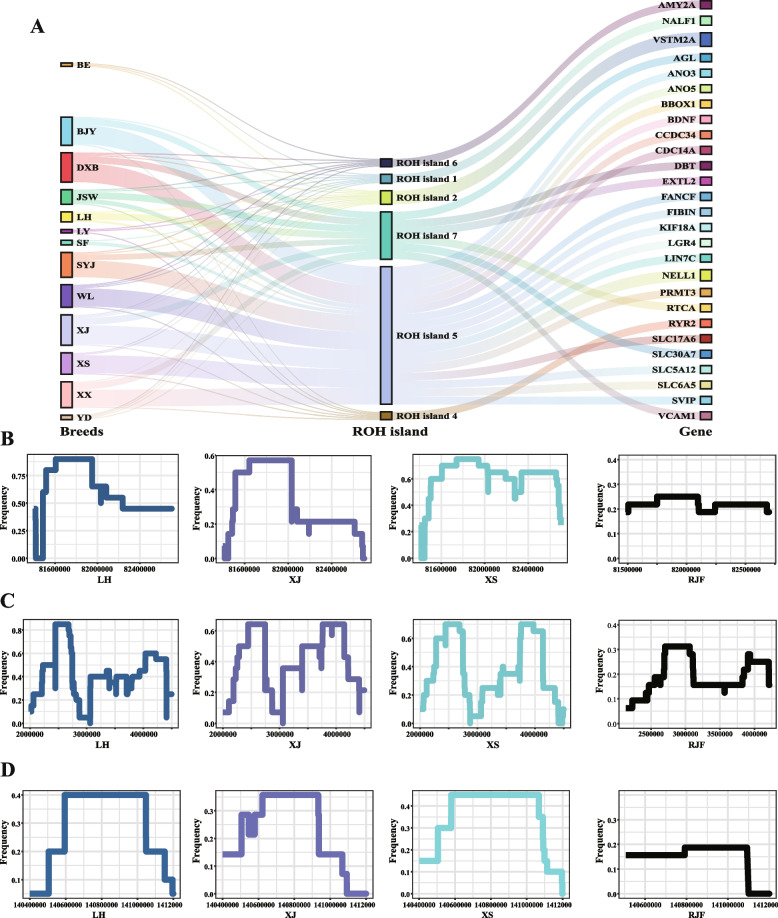
Common ROH islands in 13 indigenous chickens. **A** Candidate genes identified by 7 common ROH islands. **B**-**D** SNP frequency of VSTM2A, NELL1, and NALF1 in LH, XJ, XS, and RJF populations, respectively. LH, Luhua chickens; XJ, Xianju chickens; XS, Xiaoshan chickens; RJF, red jungle fowl

As mentioned above, the ROH islands detected in more than 7 chicken populations were defined as common ROH islands (Fig. 3A, Table 3). The 27 genes annotated based on the 7 common ROH islands were regarded as candidate genes (Fig. 4A, Table S7). The frequency of SNPs forming ROH islands in the genes *VSTM2A*, *NELL1*, and *NALF1* is shown in Fig. 4B-D, and significant peaks were observed in the genome. *VSTM2A* was the top priority gene and was detected in all populations except for JSW chickens (Fig. 4B, Fig. S7). *NELL1* was also found to be an important candidate gene in most chickens, except for the Yandang chickens (YD), BE, and LY (Fig. 4C, Fig. S8). And similar results were detected for *NALF1* gene in XJ, XS and other populations except for Songyang Jin chickens (SYJ), BJY, LH, SF, and XX (Fig. 4D, Fig. S9). The SNP frequency profiles of other genes are provided in Table S8. To determine whether these regions were under selection or domestication, similar results from RJF chickens were incorporated into the comparison. No obvious peak (*VSTM2A*: 0.19 ~ 0.25, *NELL1*: 0.06 ~ 0.31, *NALF1*: 0 ~ 0.19) was found in the genome of RJF chickens (Fig. 4B-D), indicating that these genomic regions harboring ROH islands have been selected or are under domestication in most of the chicken breeds considered herein.

**Table 3:** Common ROH islands in 13 indigenous chickens

ROH island	Chromosome	Start	End	Enriched populations^a^
ROH island 1	1	140,652,668	141,070,499	JSW, YD, DXB, XS, LY, XJ, WL, BE
ROH island 2	2	81,476,756	81,920,993	XS, SY, DXB, XX, WL, SYJ, XJ, LH, BE, BJY, YD, LY
ROH island 3	2	86,273,420	86,612,443	BJY, XS, DXB, JSW, XX, WL, SF, XJ
ROH island 4	3	36,384,718	37,208,264	BE, BJY, DXB, JSW, LH, LY, SF, WL, XJ, XS, XX, YD
ROH island 5	5	2,296,258	4,105,842	BJY, DXB, JWS, LH, SF, SYJ, WL, XJ, XS, XX
ROH island 6	8	55,919	272,482	BE, DXB, JSW, SYJ, WL, XJ, YD
ROH island 7	8	11,475,421	11,838,824	BJY, DXB, JSW, LH, SYJ, XJ, XX, YD

^a^*BE* Baier chicken, *BJY* Beijing You chicken, *DXB* Dongxiang Black chicken, *JSW* Jiangshan white-feathered chicken, *LH* Luhua chicken, *LY* Longyou chicken, *SF* Silkie feather chicken, *SYJ* Songyang Jin chicken, *WL* Wenling chicken, *XJ* Xianju chicken, *XS* Xiaoshan chicken, XX Xiaoxiang chicken, *YD* Yandang chicken

### Selective sweep analysis within ROH islands

**Fig. 5:**
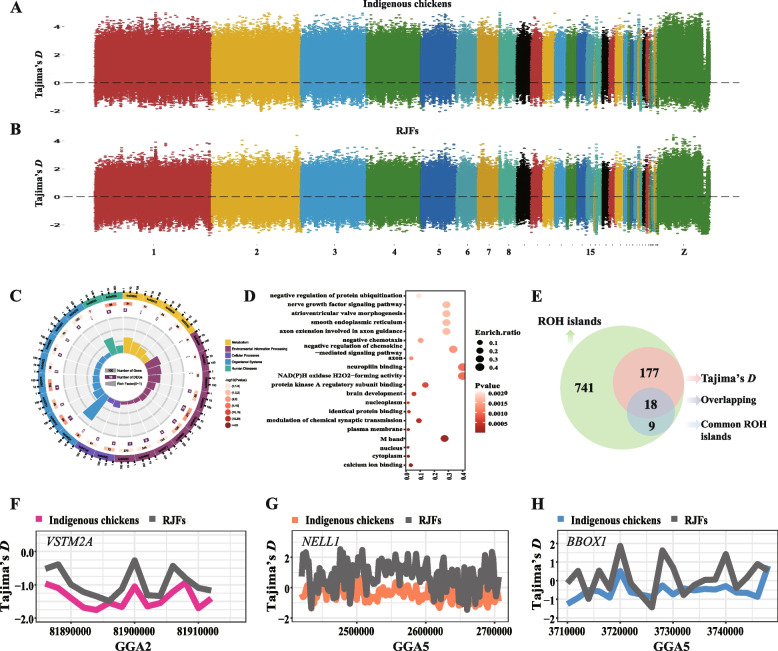
Selective signatures of ROH islands. **A**-**B** Tajima’s D index in indigenous chickens and RJFs, respectively. **C**-**D** KEGG and GO enrichment based on the selected genes. **E** Venn plot for the relationship of genes detected by ROH islands and Tajima’s D. **F**–**H** Tajima’s D results for VSTM2A, NELL1, and BBOX1 genes in indigenous chickens and RJFs. RJF, red jungle fowl

To evaluate the selection signatures of ROH islands in indigenous chickens, we calculated the Tajima’s *D* index with a window of 2 kb under the ROH islands and compared it with those of indigenous chickens and RJF. A total of 2,124 windows were detected as being under selection (Table S9), and these windows were mapped to 195 protein-coding genes (Fig. 5A-B, Table S10). The enrichment results showed that the GnRH signaling pathway, MAPK signaling pathway, starch and sucrose metabolism, and others were significantly enriched (Fig. 5C, Table S11). Calcium ion binding function was the most prominent function based on GO enrichment (Fig. 5D, Table S12). Additionally, the overlapping results showed that 18 genes presented a high ROH island incidence and low Tajima’s *D* values, including *VSTM2A*, *NELL1*, and *RYR2*. (Fig. 5E-H, Table S13). The top priority gene based on ROH island incidence, *NALF1*, was not found to have a selective signature compared to that of RJF (Fig. S10).

### Expression profile for candidate genes in indigenous chickens

**Fig. 6:**
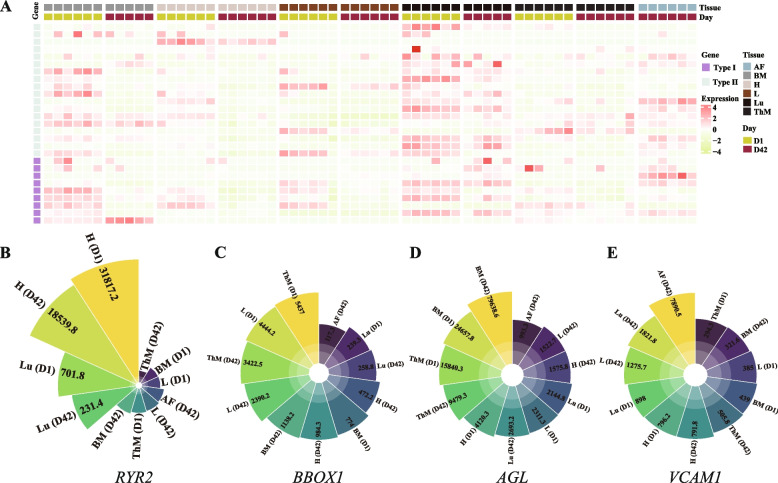
Expression profile for 27 candidate genes. **A** Expression atlas for 27 candidate genes at different stages and tissues. Type I indicated the genes detected by both common ROH islands and Tajima’s D, while type II indicated the genes detected by common ROH islands (**B**-**E**) Polar bar plot of gene expression of RYR2, BBOX1, AGL, and VCAM1 in different tissues. D1, day 1; D42, day 42; AF, abdominal fat; BM, breast muscle; H, heart; L, liver; Lu, lung; ThM, thigh muscle

For the 18 candidate genes, we calculated gene expression values in six tissues of BJY chickens (Table S14). However, the top priority genes, *VSTM2A*, *NELL1*, *SLC6A5*, and *SLC5A12*, were not expressed in these tissues, nor was the *NALF1* gene, which may function in other developmental stages or tissues (Fig. 6A). The *RYR2* gene was specifically expressed in the heart which was consistent with the biological function of heart regulation (Fig. 6B). *BBOX1* was widely expressed in multiple tissues, but a higher transcription level of *BBOX1* was detected in the liver and thigh muscle in two developmental stages (Fig. 6C). For the genes with high ROH islands incidence, we found that the glycogen metabolism related gene *AGL* was highly expressed in these tissues, especially in breast muscle (Fig. 6D). *VCAM1* was also expressed in various tissues, and higher *VCAM1* expression was found in abdominal fat tissue (Fig. 6E).

## Discussion

Chickens are efficient and food-saving farm animals that provide the largest share of meat and egg products consumed by humans [40]. Compared to commercial chickens, the meat and eggs of indigenous chickens are of better quality but are produced less efficiently [41–43]. The fundamental causes of this low efficiency are low selection intensity [6], limited studies involving genetic resource exploration, and failure to continuously develop characteristic traits. In fact, there is a lack of unified assessment standards for the evaluation of genetic resource conservation effects, and it is difficult to measure the purity of consanguinity, the level of population inbreeding and the genetic diversity of indigenous chickens. However, this information is of great significance for breed protection, biodiversity and new chicken breed/strain cultivation. ROH and a series of inbreeding coefficients (F_HOM_, F_GRM_, F_UNI_, F_ROH_) calculated based on genome-wide SNPs allow a precise assessment for genetic resource conservation [13, 44–46]. In this study, we assessed the conservation effect in 13 indigenous chicken breeds based on ROH and inbreeding status, and we evaluated the selection effect around the ROH island based on genome-wide high-quality variants.

Genomic heterozygosity (Ho and He) is associated with population diversity, and the calculation of results using genome-wide SNPs can avoid bias [47, 48]. We observed that Ho and He were both close to 0.3 in all chickens, which was consistent with the reports of Yuan et al. and Liu et al. [49]. SF chickens had the highest heterozygosity (Ho = 0.32, He = 0.31), while WL chickens presented low heterozygosity (Ho = 0.26, He = 0.27), indicating that relatively intensive selection has occurred in WL chickens. Higher heterozygosity was observed than expected in all populations except for WL chickens. This indicates a modern bottleneck in these populations, which could be caused by selection or domestication [50]. Compared to pedigree information, genome-wide SNPs are more accurate for assessing inbreeding and relatedness across populations [13]. F_GRM_, F_HOM_, and F_UNI_ are all based on the principle of identity by status, which cannot distinguishes ambiguity between identity by status and identity by descent and is influenced by allele frequency [12, 45]. F_ROH_ is affected by the ratio of long homozygous segments to whole-genome length and is less impacted by the quality of experimental materials and DNA samples [51]. This increases the accuracy of inbreeding coefficient calculation based on ROH in humans [7], cattle [9], pigs [10], sheep and goats [11, 52], etc. In this study, the inbreeding coefficients among the 13 populations indicated that F_ROH_ generally presented a lower value than the other indices. A favorable conservation effect was found in all chicken breeds (F_ROH_ = 0.02 ~ 0.13) except for LH chickens (F_ROH_ = 0.24). Therefore, indigenous chickens are less influenced by inbreeding and selection, and considerable genetic diversity is harbored within these populations. This is consistent with results reported in Tibetan chickens [14]. Additionally, a high correlation between F_ROH_ and other indices (F_GRM_, F_HOM_, and F_UNI_) was found, which was highly consistent with previous reports in modern chickens and other farm animals [53–56]. This also proved that F_ROH_ is more accurate in assessing inbreeding status based on identity by descent [57].

ROH is affected by the length of homozygous segments in the genome, and ROH length is usually less than 1 Mb. In Zhang et al.’s study, the ROH lengths of BJY, BE, and Langshan chickens were less than 200 kb and were maintained in multiple generations [18]. In modern chickens, Talebi et al. revealed that > 50% ROH were focused in the length category of less than 1 Mb, especially in broilers (> 70%) [53]. Here, we calculated statistics of the distribution of ROH. The number of ROH ranged from 79.0 to 444.2, and 67.9% ~ 89.9% of ROH had a length of < 1 Mb. This was highly consistent with previous reports [12, 14]. In addition, directed changes in allele frequency could be caused by artificial selection or domestication, which could also lead to the occurrence of homozygous segments with different lengths [58–60]. Therefore, ROH, especially ROH islands, are usually focused on selected regions, and these regions and surrounding genes are related to specific traits [60]. Here, we detected ROH islands in each studied population and annotated them using the reference genome and QTLdb. Body weight is the most important economic trait based on QTL annotation, consistent with the goal of selective breeding and similar to the findings of Talebi et al. [53]. Among the 13 indigenous chicken breeds, a total of 48 ~ 495 genes were found in corresponding ROH islands. The starch and sucrose metabolism was enriched in 7 populations, and lipid storage was significantly changed in all populations except JSW. Huang et al. proved that microbial starch and sucrose metabolism was highly correlated with feed efficiency in chickens [61]. This finding implies a potential regulatory target for improving feed efficiency in the breeding system of indigenous chickens.

For these ROH islands among 13 populations, the common ROH islands are summarized. We found that ROH islands covering *VSTM2A* and *NELL1* were identified in more than 10 populations. The *NALF1* gene was also annotated by ROH islands in 8 populations. Additionally, to validate selection status within ROH islands, we calculated the Tajima’s *D* index in indigenous chickens and RJF. We found that 19 genes (e.g., *VSTM2A*, *NELL1*, and *BBOX1*) were both selected and covered by ROH islands. VSTM2A is secreted in committed preadipocytes and produced to preserve and amplify the adipogenic capability of adipose precursors [62]. This gene played an important role in the embryonic stage, whereas we found little expression of the gene in abdominal fat and other tissues in BJY chickens. *NELL1* is a putative functional gene associated with skeletal integrity in chickens [63], but no expression of this gene was found in skeletal muscle. These results indicate that *NELL1* could function in other developmental stages (e.g., embryonic stage). For the *RYR2* gene, Fedorova et al. found that it was correlated with adaptation to cold environments and covered by an ROH island in Russian White chickens [17]. *RYR2* was highly correlated with heart rate, consistent with the expression characteristics observed in this study. *BBOX1* plays a crucial role in mitochondrial beta-oxidation [64], and a higher transcription level was detected in liver and thigh muscle in two developmental stages in this study. Therefore, we suggest that *VSTM2A* could putatively function in chicken domestication or artificial selection.

## Conclusions

In this study, whole-genome sequencing was used to capture variants and assess genomic heterozygosity and inbreeding and to investigate ROH and putative selected genes in 13 chicken breeds. Heterozygosity in these 13 populations ranged from 0.26 to 0.32, and LH chickens were more affected by inbreeding and exhibited the maximum number of ROH. A total of 291 ROH islands covering 2,321 known QTLs and 945 genes were identified. The starch and sucrose metabolism was significantly different in 7 populations. Combined analysis with the selective sweep results suggested genes such as *VSTM2A*, *NELL1*, and *RYR2* as putative candidate genes for human selection or domestication. Our findings facilitate the understanding of population inbreeding and may provide potential candidate markers for selection and domestication, which could contribute to the design and implementation of modern breeding and conservation strategies for chickens.

### Supplementary Information

**Supplementary Material 1.**

**Supplementary Material 2.**

## Data Availability

The whole-genome sequencing data of 209 individuals is available from NCBI Sequence Read Archive and have been assigned BioProject accession PRJNA942350 (https://www.ncbi.nlm.nih.gov/sra/?term=PRJNA942350). All data generated from this study are available from the supplemental materials and the corresponding author on reasonable request.

## Abbreviations

BE: Baier chickens
BJY: Beijing You chickens
DXB: Dongxiang Black chickens
Ho: Observed heterozygosity
He: Expected heterozygosity
IBS: Identity-by-state
JSW: Jiangshan white-feathered chickens
LH: Luhua chickens
LY: Longyou chickens
QTL: Quantitative trait loci
RJF: Red jungle fowl
ROH: Runs of homozygosity
SF: Silkie feather chickens
SNP: Single nucleotide polymorphism
SYJ: Songyang Jin chickens
WL: Wenling chickens
XJ: Xianju chickens
XS: Xiaoshan chickens
XX: Xiaoxiang chickens
YD: Yandang chickens
