# Supplementary material for: Genome-wide detections for runs of homozygosity and selective signatures reveal novel candidate genes under domestication in chickens
Source: BMC Genomics. 2024 May 16;25:485. doi: 10.1186/s12864-024-10349-4 (PMC11097469; doi:10.1186/s12864-024-10349-4)
Supplement: Supplementary file 1 — Supplementary Material 1. [file 12864_2024_10349_MOESM1_ESM.docx]

**Supplementary Material**

**Genome-wide detections for runs of homozygosity and selective signatures reveal novel candidate genes under domestication in chickens**

Xiaodong Tan ^a,1^, Lu Liu ^b,c,1^, Jie Dong ^a^, Minjie Huang ^a^, Jiawen Zhang ^a^, Qinghai Li ^d^, Huanhuan Wang ^d^, Lijuan Bai ^e^, Ming Cui ^e^, Zhenzhen Zhou ^a^, De Wu ^f^, Yun Xiang ^c,^*, Weifen Li ^b,^*, Deqian Wang ^a,^*

^a^ Institute of Animal Husbandry and Veterinary Science, Zhejiang Academy of Agricultural Sciences, Hangzhou 310021, China.

^b^ College of Animal Sciences, Zhejiang University, Hangzhou, Zhejiang, 310058, China.

^c^ Jinhua Jinfan Feed Co., Ltd, Jinhua, Zhejiang, 321000, China.

^d^ Animal Husbandry Institute, Hangzhou Academy of Agricultural Sciences, Hangzhou 310024, China

^e^ Zhejiang Animal Husbandry Technology Extension and Breeding Livestock and Poultry Monitoring Station, Hangzhou 310020, China

^f^ Postdoctoral Research Station, Jinhua Development Zone, Jinhua, Zhejiang 321000, China

^1^ These authors contributed equally to this work.

^*^ Corresponding author: Yun Xiang, Jinhua Jinfan Feed Co., Ltd, Jinhua, Zhejiang, 321000, China, [xyjhzj@aliyun.com](mailto:xyjhzj@aliyun.com); Weifen Li, College of Animal Sciences, Zhejiang University, Hangzhou, Zhejiang, 310058, China, [wfli@zju.edu.cn](mailto:wfli@zju.edu.cn); Deqian Wang, Institute of Animal Husbandry and Veterinary Science, Zhejiang Academy of Agricultural Sciences, Hangzhou 310021, China, [wangdq@zaas.ac.cn](mailto:wangdq@zaas.ac.cn).


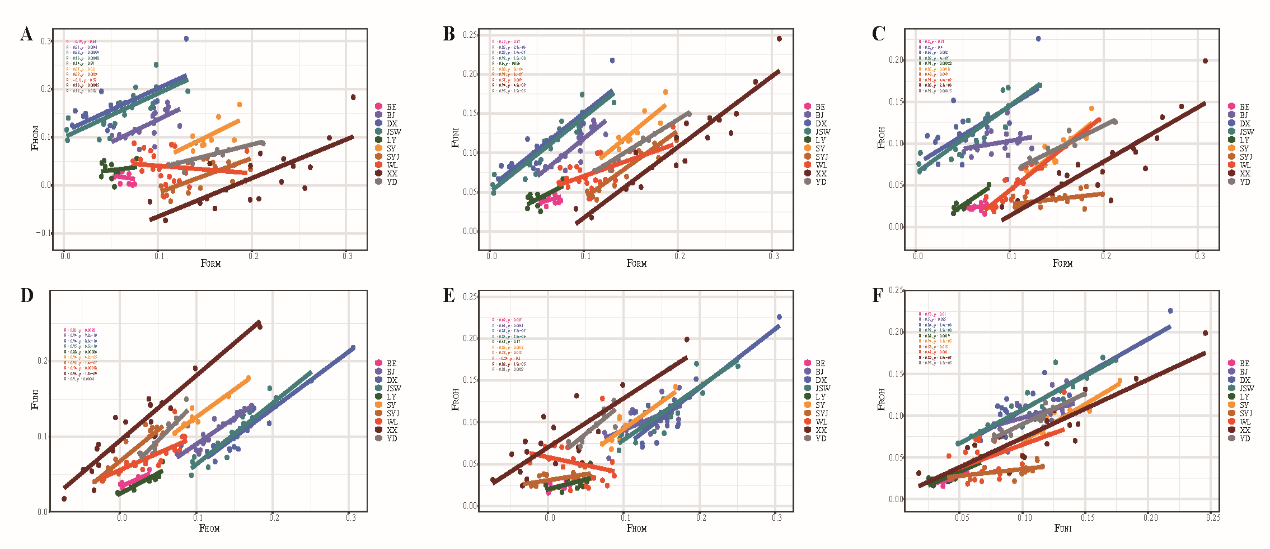


Fig. S1 Correlations among 4 genomic inbreeding in each population.


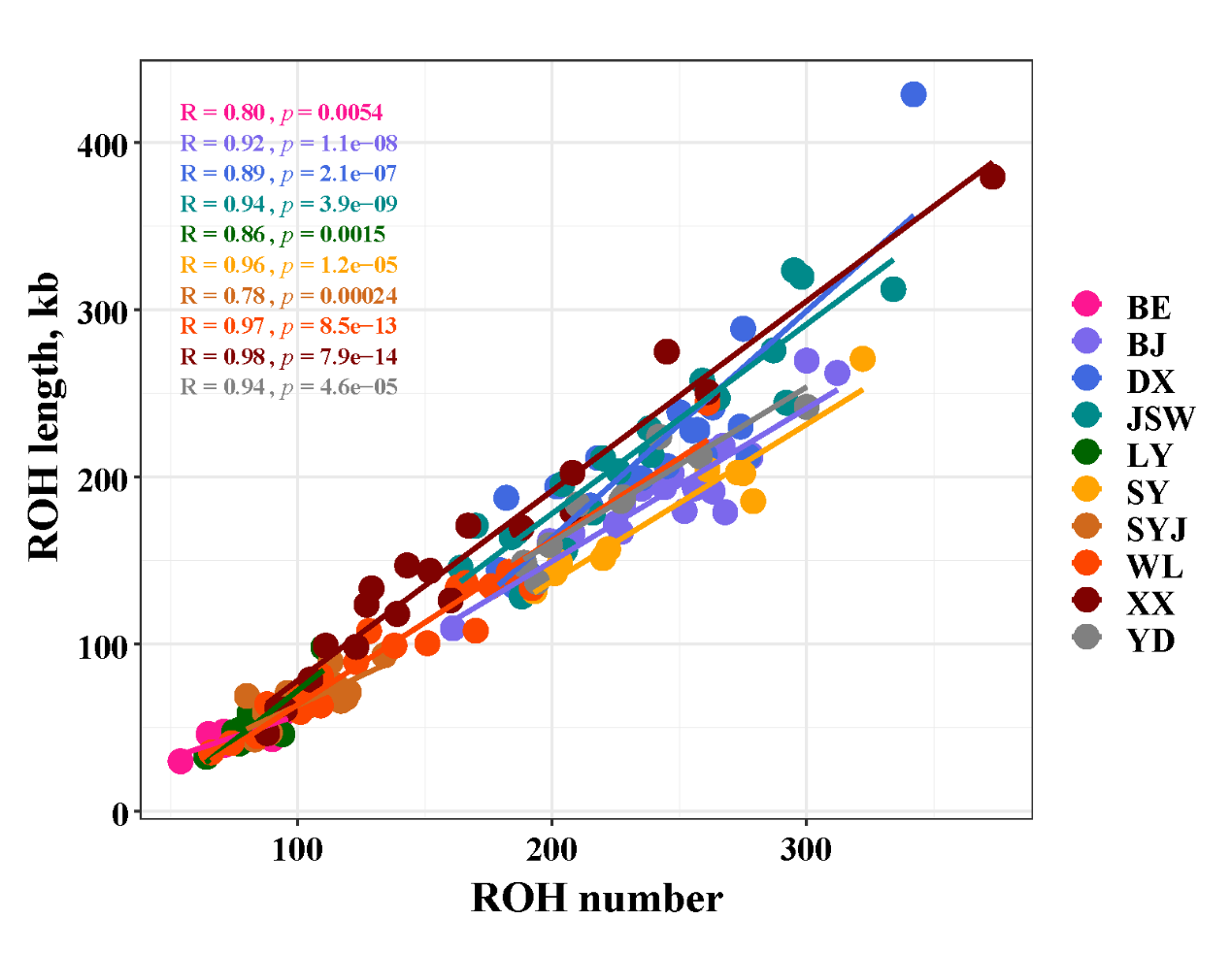
Fig. S2 Correlation between ROH length and number in each population.

**
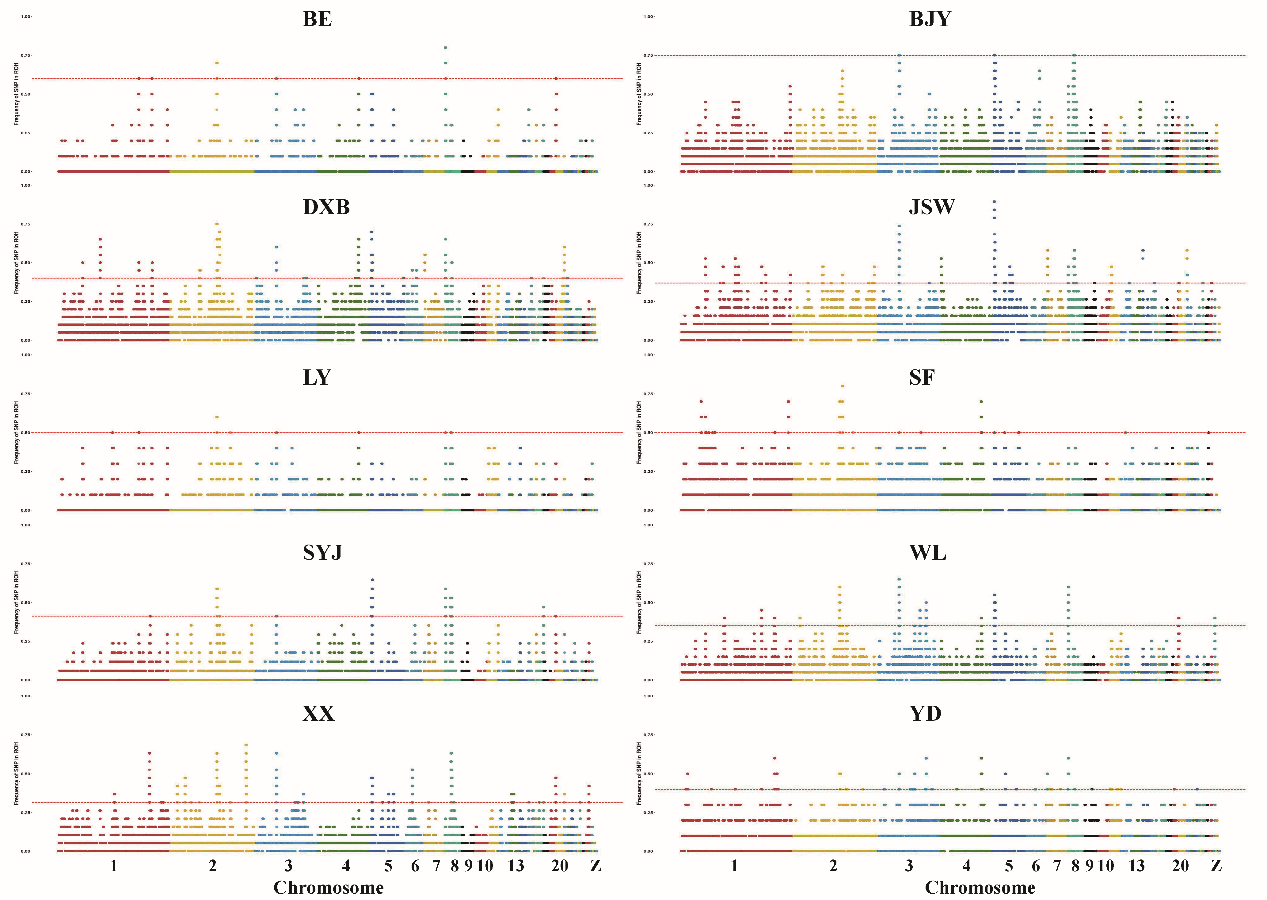
**Fig. S3 Detection of whole-genome ROH islands in each population


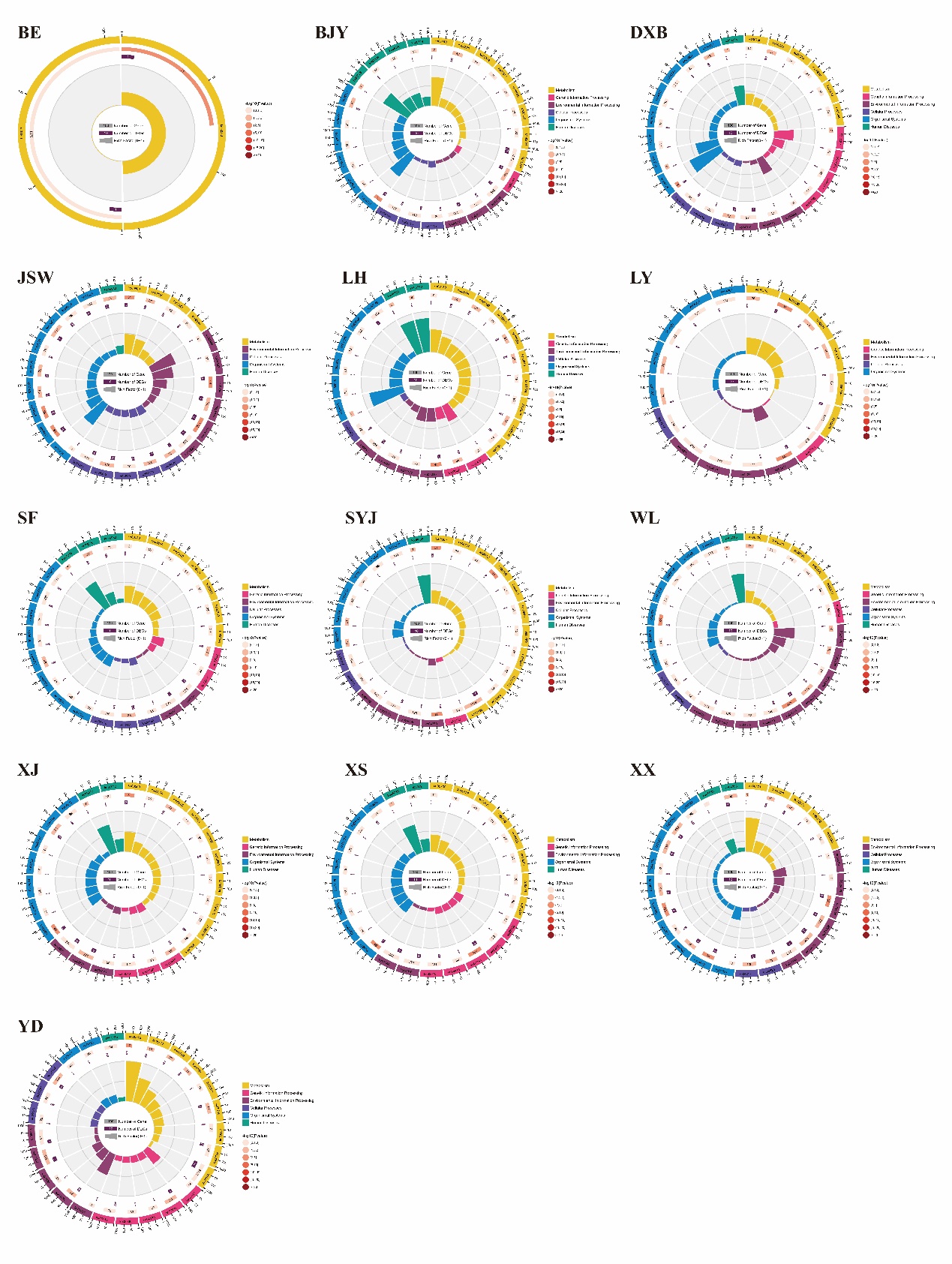
Fig. S4 KEGG enrichment based on the genes covered by ROH islands.


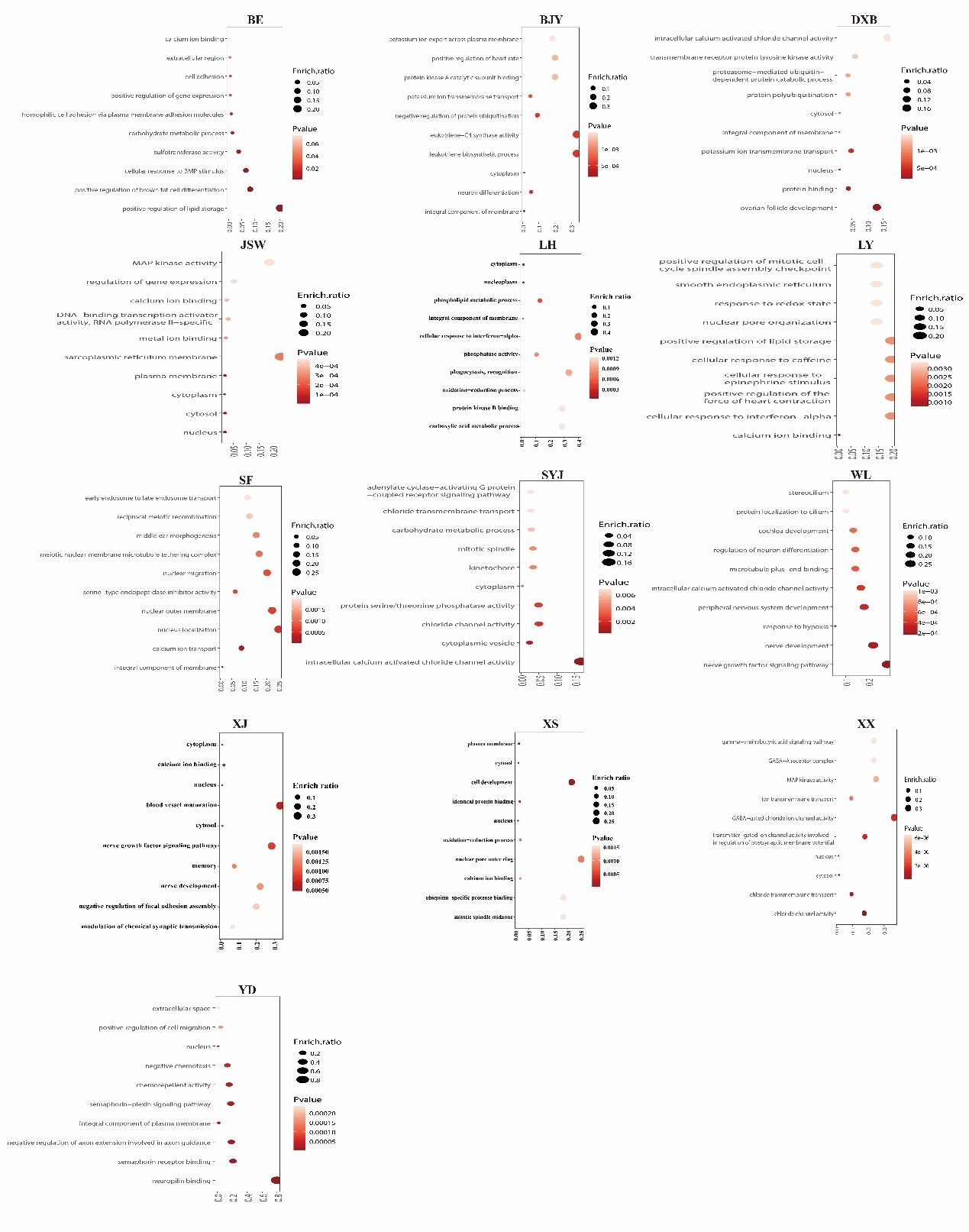
Fig. S5 GO enrichment based on the genes covered by ROH islands.

**
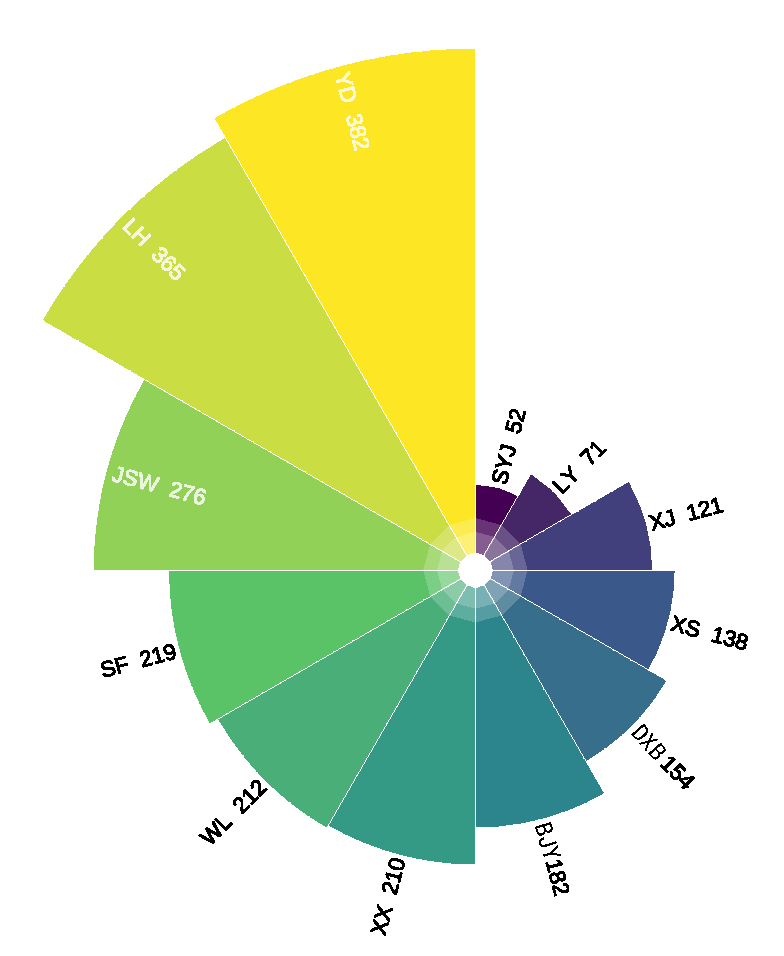
**

Fig. S6 Number of known QTLs mapped by ROH islands in each population.


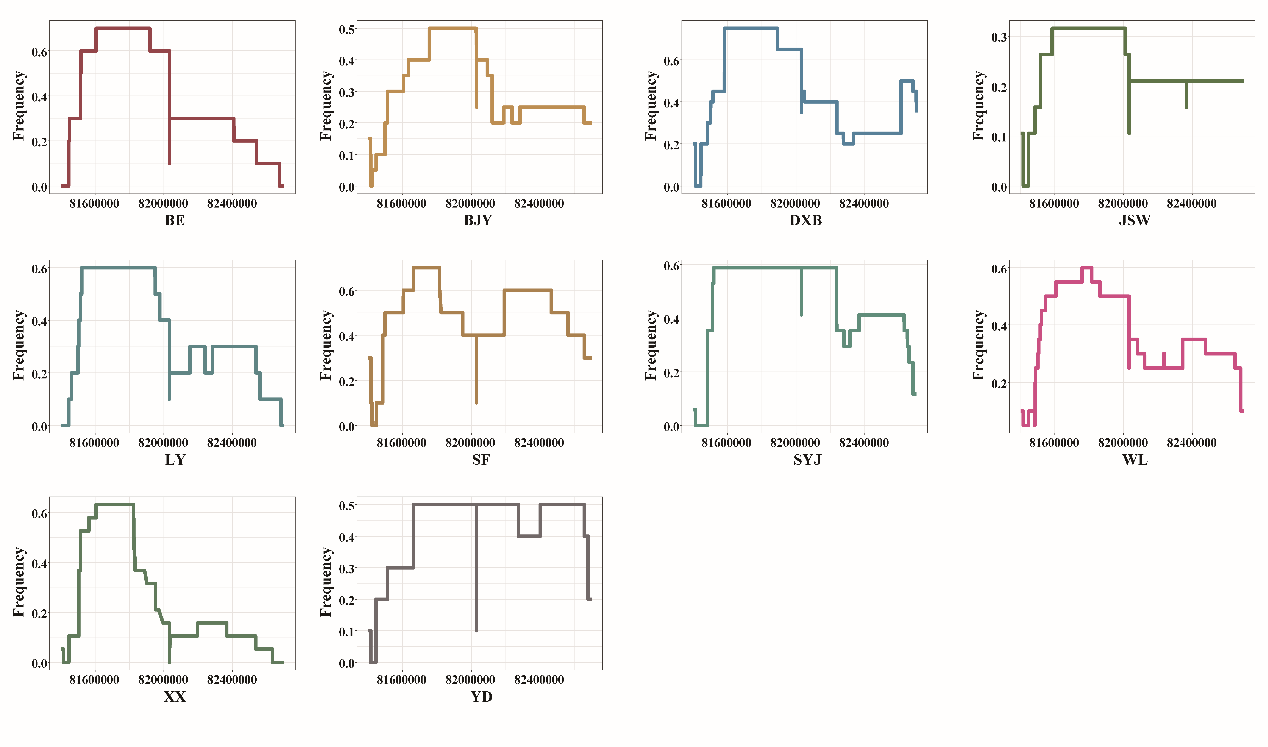
Fig. S7 SNP frequency within *VSTM2A* gene.

**
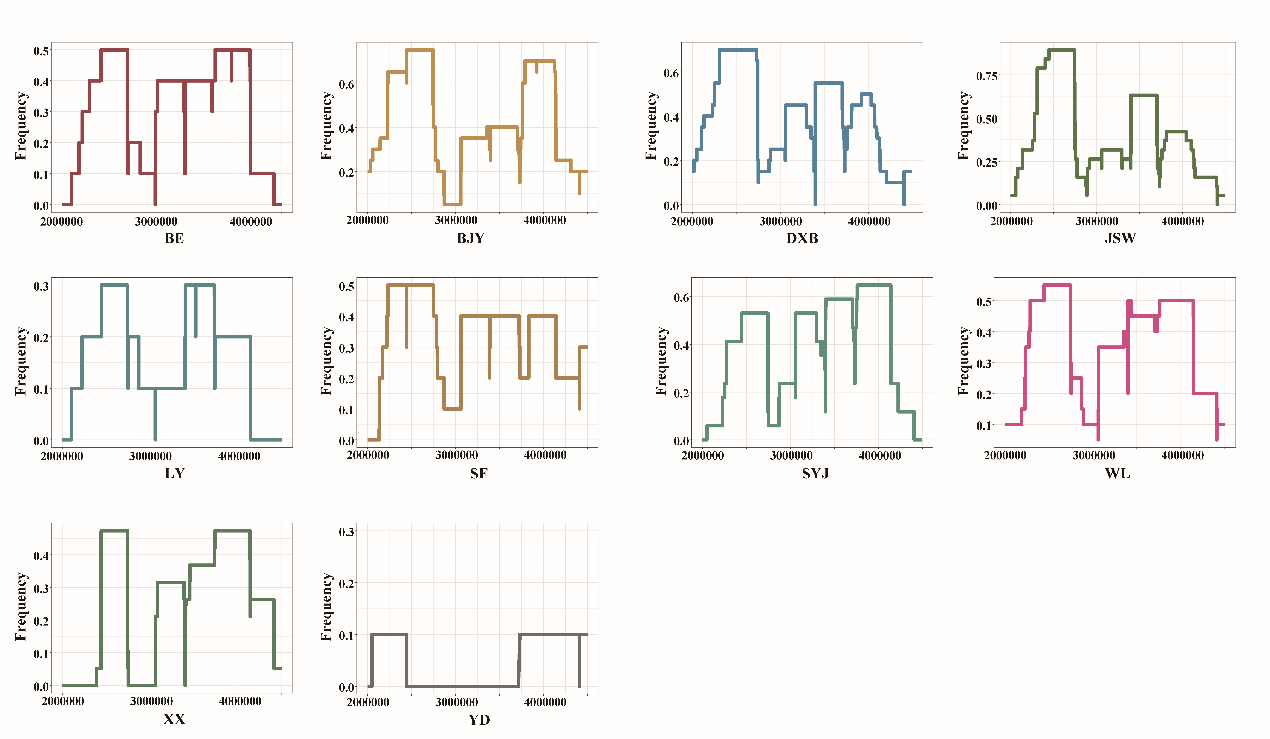
**Fig. S8 SNP frequency within NELL1 gene.

**
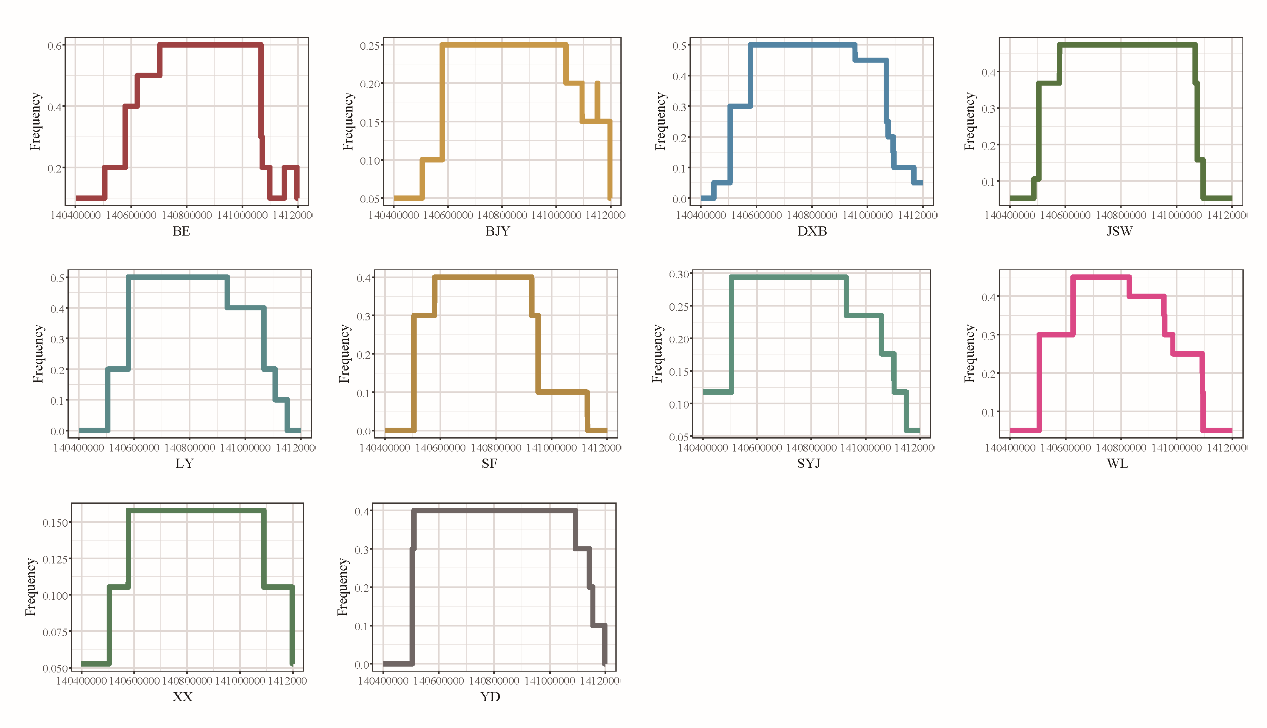
**Fig. S9 SNP frequency within *NALF1* gene.

**
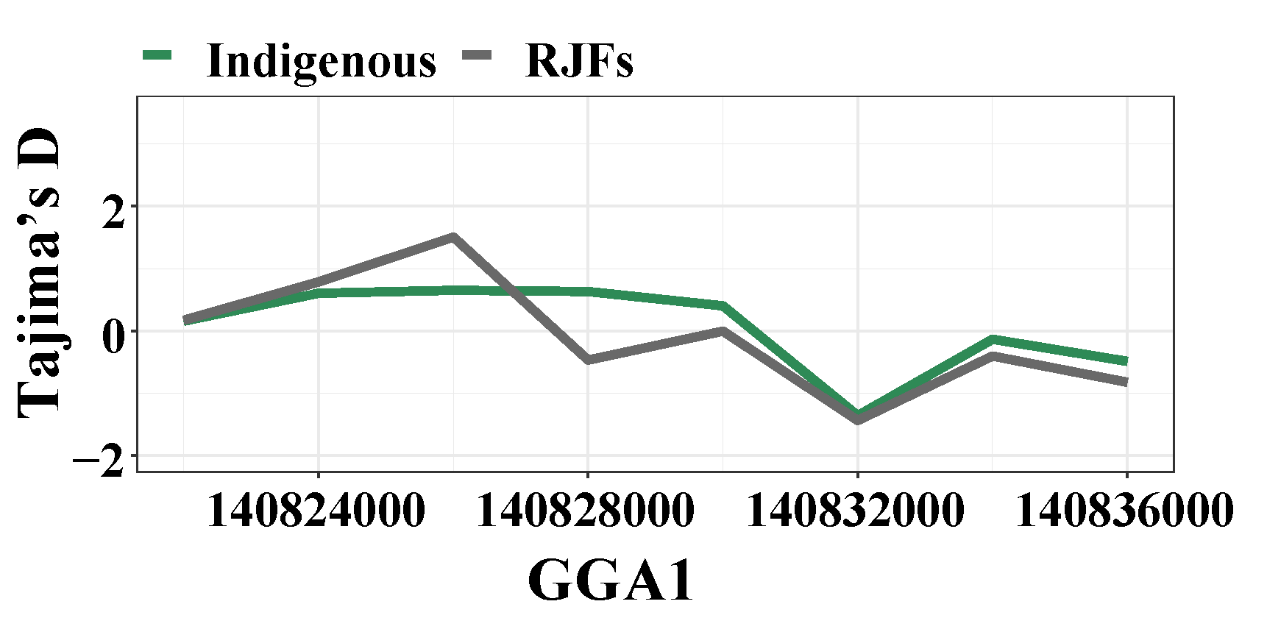
**Fig. S10 Tajima’s *D* value in *NALF1* gene in indigenous chickens and RJFs.
